# Tourism Demand Forecast Based on Adaptive Neural Network Technology in Business Intelligence

**DOI:** 10.1155/2022/3376296

**Published:** 2022-01-18

**Authors:** Liangliang Wang

**Affiliations:** Xinyang Agriculture and Forestry University, Xinyang 464000, China

## Abstract

In order to improve the effect of tourism demand forecast, the commercial development of the tourism industry, and the actual experience of users, this paper uses adaptive neural network technology to conduct tourism demand forecast analysis. Moreover, this paper improves the adaptive neural network algorithm so that it can handle multiple data for tourism demand forecast. After improving the algorithm, this paper employs the actual process of tourism demand forecast to construct a tourism demand forecast model based on adaptive neural network technology. After that, this paper combines travel time and space data analysis to determine the system's functional structure and network topology. Through experimental research, it can be seen that the tourism demand forecast model based on adaptive neural network technology proposed in this paper performs well in tourism demand forecast and meets the actual demand of modern tourism forecast.

## 1. Introduction

Since the beginning of the 21st century, the world's tourism industry has developed rapidly. Currently, many cities regard tourism as the pillar industry of their economic development, hoping to use the development of tourism to drive the development of the entire social economy. An obvious difference between tourism and industry, agriculture, and other production industries is the volatility of tourism, which is a huge challenge for enterprises and government planning departments in the tourism market. Therefore, in order to make a successful decision, we need to make an accurate forecast of the future demand of the tourism market. Modern tourism researchers have tried to use many mathematical methods and models to solve this problem. The main models commonly used at present are time series forecast models; regression models, including linear and nonlinear regression models; adaptive models; artificial neural networks; etc.

As a “green” driving force, tourism is not only related to the balance and coordination of resources and the environment, but also one of the important ways to achieve sustainable economic development [1]. Since the reform and opening up, the development of China's tourism industry has experienced a historical leap from “shortage type” to “large country type.” However, with the rapid growth of the tourism industry, the number of complaints about tourism experience is increasing, and the negative information exposed by the media and tourists through the Internet has a serious impact on the image of regional tourism [2]. In the Internet age, the dissemination of these negative information spans traditional geographic distance and time constraints [3] and has attracted more and more attention. Among them, the media and tourists' online attention to negative information such as travel complaints has become an important factor influencing tourists' decision-making and the development of regional tourism. At the same time, the massive amount of network information has made tourism routes more diverse, and the spatial associations between tourism demand-oriented regional tourism industries have become increasingly close and complex. Therefore, the spatial spillover effect of negative information network attention on regional tourism development cannot be ignored.

Some scholars divide the influencing factors into three types: thrust, pull, and resistance. Among them, push refers to the factors that encourage people to travel, including population size, income trends, income distribution, and education level. Rally refers to the factors that attract people's subjective wishes to travel to a certain area, including climate, business, culture, marketing strategies, relatives, and friends. Resistance refers to the factors that hinder people from traveling to a certain area, including price, competitors, and supply capacity. When foreign countries use quantitative models to analyze influencing factors, they often take economic factors as the main research object. In the process of research, some scholars have discovered that the most critical factor affecting tourism demand and related decision-making is economy. Domestic scholars have also conducted research on the tourism demand of the source country of Hong Kong, showing that the tourism products, the economic level of the region, and the tourism costs are the most important factors. Special events include economic and noneconomic factors, such as social conflicts, sports events, and financial crises. In these three categories, it is generally believed that the influence of noneconomic factors is relatively small and that economic factors are more conducive to the establishment of tourism demand forecasting models; in-depth research was conducted on this basis. However, in terms of the actual situation in my country, my country's tourism demand is also greatly affected by family consumption habits, political and social factors, consumption concepts, and other factors. Therefore, the tourism demand forecasting method that only considers economic factors is not in line with my country's reality.

This paper uses adaptive neural network technology to carry out tourism demand forecast and analysis and build a business intelligence model to improve the forecast effect of subsequent tourism demand.

## 2. Related Work

Many scholars try to use different models to improve the accuracy and timeliness of demand forecasting, which can usually be divided into three categories: causality model, time series model, and artificial intelligence model. Literature [4] uses search keywords to establish a multiple linear regression function to predict the number of daily tourists and uses a single variable autoregressive moving average (ARMA) model to predict the tourism demand of 9 countries in the Asia-Pacific region. Sigalat Signes et al. [5] constructed a combined prediction model of autoregressive moving average model and BP neural network. Literature [6] applies support vector regression (SVR) model to tourism demand forecasting and combines it with autoregressive integrated moving average (ARIMA) model. Literature [7] uses an artificial neural network model to predict tourism demand in the Balearic Islands. Literature [8] uses a neural network-based fuzzy time series model as a prediction model to predict tourism demand during SARS. Literature [9] proposes a tourism flow forecasting SSVR-PSO (seasonal support vector regression particle swarm optimization) model that combines the seasonal support vector regression model and particle swarm optimization algorithm. Compared with the first two types of models, artificial intelligence models show great advantages in terms of nonlinear fitting and adaptive learning, but traditional artificial neural networks have problems such as complex training process and long-time consumption. Therefore, finding efficient and accurate new neural network methods has become a hot research direction. Paper [9] proposed ESN (echo state network). Compared with traditional algorithms, the training process of echo state network is simple and efficient. It can approximate dynamic systems infinitely under general conditions and is widely used in various prediction problems. Literature [10] uses ESN to predict mobile communication traffic, and literature [11] uses it to deal with time series problems related to the prediction of certain eigenvalues of sunspots. However, ESN also has its own shortcomings and limitations. For example, ESN parameters and connection weights are randomly set, and only the output layer weights are adjusted during the training process, which is easy to fall into the local optimal solution. The common method currently used is to construct the ESN model when the network selects the optimal output multiple times, or select the parameters based on experience to construct the model. In this regard, many scholars have optimized the structure of the ESN. Literature [12] developed several new storage pool topologies for ESN and predicted the energy consumption of office buildings. Literature [13] proposed an echo state network prediction model with bifurcation structure to improve the prediction accuracy of ESN. Some scholars also use heuristic algorithms to optimize the parameters of ESN and obtain good experimental results. Literature [14] uses genetic algorithms to optimize the initial weights and thresholds of the forward feedback neural network, but there are relatively few studies of this kind at present. The fruit fly optimization algorithm (FOA) proposed by literature [15] has the advantages of low complexity, fast calculation speed, and strong solution ability. It has been applied in many combinatorial optimization and continuous optimization fields. Literature [16] uses FOA optimization for the structural parameters of the artificial neural network (ANN) model, and literature [14] uses FOA to optimize the penalty parameters and conversion coefficients of SVR and has achieved good experimental results. FOA itself has disadvantages such as low convergence accuracy and being easy to fall into local extreme values. Literature [17] proposed an adaptive mutation fruit fly optimization algorithm, according to the population fitness variance and the current optimal value; under the probability P, the mutation operator interferes with the replication to continue the optimization. Literature [18] increases the inertia to change the nonlinear decreasing characteristics and the relationship between individuals and groups, and an improved fruit fly optimization algorithm (IFOA) is constructed.

## 3. Tourism Demand Forecast Algorithm Based on Adaptive Neural Network

In order to achieve the control goal, the dynamic error system is defined as follows:

(1) $$\begin{array}{c} {\tilde{x}}_{1}=x_{1}-y\;d,\\ {\tilde{x}}_{2}={\dot{\tilde{x}}}_{1},\\ {\tilde{x}}_{3}={\dot{\tilde{x}}}_{2.} \end{array}$$

The design method of linear filter reduction is adopted, and filter variables are introduced to reduce the order of the system. The linear filter is defined as follows. The filter has the properties of exponential convergence, asymptotic convergence, and bounded transfer.

(2) $$\begin{array}{c} e1={\tilde{x}}_{1},\\ e2={\dot{e}}_{1}+2e_{1},\\ e3={\dot{e}}_{2}+2e_{2}. \end{array}$$

We define $e={\left[e1^{T},e2^{T},e3^{T}\right]}^{T},\tilde{x}={\left[\tilde{x}1^{T},\tilde{x}2^{T},\tilde{x}3^{T}\right]}^{T},$ and then there is the following relationship between *e* and $\tilde{x}$:

(3) $$\begin{array}{c} e=\left[\left(\begin{array}{ccc} I_{2} & 0 & 0\\ 2I_{2} & I_{2} & 0\\ 4I_{3} & I_{3} & I_{3} \end{array}\right)\right]\tilde{x}. \end{array}$$

In order to obtain the closed-loop dynamic equations of the three channels, the following equations are obtained by finding the first-order time derivative of *e*3(*t*):

(4) $$\begin{array}{c} {\dot{e}}_{3}=\frac{d}{dt}\left({\tilde{x}}_{3}+4{\tilde{x}}_{2}+4{\tilde{x}}_{1}\right)\\ =\dot{\tilde{x}}3+4{\dot{\tilde{x}}}_{2}+4{\dot{\tilde{x}}}_{1}\\ =\dddot{\tilde{x}}1-\dddot{\tilde{y}}d+4{\tilde{x}}_{3}+4{\tilde{x}}_{2}\\ =F\left(x_{1},x_{2},x_{3}\right)+G\left(x_{1},x_{2}\right)u-\dddot{y}d+4{\tilde{x}}_{3}+4{\tilde{x}}_{2.} \end{array}$$

By decomposing formula (4), *G*(*x*_1_, *x*_2_), the following expression is obtained:

(5) $$\begin{array}{c} {\dot{e}}_{3}\left(t\right)=F\left(x_{1},x_{2},x_{3}\right)+S\left(x_{1},x_{2}\right)DU\left(x_{1},x_{2}\right)u-\dddot{y}d+4{\tilde{x}}_{3}+4{\tilde{x}}_{2}. \end{array}$$

By multiplying both sides of the above equation by *S*^−1^(*x*_1_, *x*_2_), we get

(6) $$\begin{array}{c} S^{-1}\left(x_{1},x_{2}\right){\dot{e}}_{3}\left(t\right)=S^{-1}\left(x_{1},x_{2}\right)F\left(x_{1},x_{2},x_{3}\right)\\ +DU\left(x_{1},x_{2}\right)u+S^{-1}\left(x_{1},x_{2}\right)\left(-\dddot{y}\text{d}+4{\tilde{x}}_{3}+4{\tilde{x}}_{2}\right). \end{array}$$

By adding and subtracting (1/2)*S*^−1^(*x*_1_, *x*_2_)*e*3 at the same time in the right half of (6), we can get

(7) $$\begin{array}{c} S^{-1}\left(x_{1},x_{2}\right){\dot{e}}_{3}\left(t\right)=S^{-1}\left(x_{1},x_{2}\right)F\left(x_{1},x_{2},x_{3}\right)+S^{-1}\left(x_{1},x_{2}\right)\left(-\dddot{y}d+4{\tilde{x}}_{3}+4{\tilde{x}}_{2}\right)\\ +DU\left(x_{1},x_{2}\right)u+\frac{1}{2}S^{-1}\left(x_{1},x_{2}\right)e3-\frac{1}{2}S^{-1}\left(x_{1},x_{2}\right)e3. \end{array}$$

Matrix *D* is a diagonal matrix, and the value of each item in the matrix is +1 or −1. The matrix form is

(8) $$\begin{array}{c} D=\left(\begin{array}{ccc} d_{1} & 0 & 0\\ 0 & d_{2} & 0\\ 0 & 0 & d_{3} \end{array}\right). \end{array}$$

The matrix *U* is an upper triangular matrix, in which each function above the main diagonal is an unknown function item. The matrix form is

(9) $$\begin{array}{c} U\left(x_{1},x_{2}\right)=\left(\begin{array}{ccc} 1 & U_{12}\left(x_{1},x_{2}\right) & U_{13}\left(x_{1},x_{2}\right)\\ 0 & 1 & U_{23}\left(x_{1},x_{2}\right)\\ 0 & 0 & 1 \end{array}\right). \end{array}$$

In order to facilitate subsequent control design, a new auxiliary function variable is designed, and the auxiliary function is defined as

(10) $$\begin{array}{c} N\left(x_{1},x_{2},x_{3},y\;d,\dot{y}d,\ddot{y}d,\dddot{y}d\right)=S^{-1}\left(x_{1},x_{2}\right)F\left(x_{1},x_{2},x_{3}\right)\\ +S^{-1}\left(x_{1},x_{2}\right)\left(-\dddot{y}d+4{\tilde{x}}_{3}+4{\tilde{x}}_{2}\right)+\frac{1}{2}S^{-1}\left(x_{1},x_{2}\right)e3. \end{array}$$

By substituting (10) into (7), the open-loop error dynamic system of *e*3(*t*) is obtained as follows:

(11) $$\begin{array}{c} S^{-1}\left(x_{1},x_{2}\right){\dot{e}}_{3}\left(t\right)=N\left(\cdot \right)+DU\left(x_{1},x_{2}\right)u-\frac{1}{2}S^{-1}\left(x_{1},x_{2}\right)e3. \end{array}$$

The hypersonic vehicle dynamics control signal is defined as Φ=*u*1, *δe*=*u*2, *δc*=*u*3. According to the closed-loop dynamics equation in (10), in order to facilitate the subsequent stability analysis process, the control signal *u*(*t*) is designed as follows:

(12) $$\begin{array}{c} u={\left[\Phi \left(t\right),\delta e\left(t\right),\delta c\left(t\right)\right]}^{T}={\left[u1,u2,u3\right]}^{T}. \end{array}$$

Here, *u*(*t*) is decomposed into

(13) $$\begin{array}{c} ui=uri+u\;di\;,i=1,2,3. \end{array}$$

*uri* is defined as follows:

(14) $$\begin{array}{c} uri=di\left(-ki\right)e3i. \end{array}$$

By substituting matrix U, matrix *D*, and the resulting equation into (14), the closed-loop error dynamic equation is obtained:

(15) $$\begin{array}{c} S^{-1}\left(x_{1},x_{2}\right){\dot{e}}_{3}\left(t\right)=\left[\begin{array}{c} N1\left(\cdot \right)\\ N2\left(\cdot \right)\\ N3\left(\cdot \right) \end{array}\right]-\frac{1}{2}{\dot{S}}^{-1}\left(x_{1},x_{2}\right)e3\\ +\left(\begin{array}{ccc} d_{1} & 0 & 0\\ 0 & d_{2} & 0\\ 0 & 0 & d_{3} \end{array}\right)\left(\begin{array}{ccc} 1 & U_{12} & U_{13}\\ 0 & 1 & U_{23}\\ 0 & 0 & 1 \end{array}\right)\left[\begin{array}{c} ur_{1}+ud_{1}\\ ur_{2}+ud_{2}\\ ur_{3}+ud_{3} \end{array}\right]. \end{array}$$

By expanding (15), we can get

(16) $$\begin{array}{c} S^{-1}\left(x_{1},x_{2}\right){\dot{e}}_{3}\left(t\right)=\left(\begin{array}{c} N1\left(\cdot \right)+d1\left(u12\text{(}ur_{2}+ud_{2}\right)+u13\left(ur_{3}+ud_{3}\right)\\ N2\left(\cdot \right)+d2\left[u23\left(ur_{3}+ud_{3}\right)\right]\\ N3\left(\cdot \right) \end{array}\right)\\ -\frac{1}{2}{\dot{S}}^{-1}\left(x_{1},x_{2}\right)e3+\left(\begin{array}{c} d1\left(ur_{1}+ud_{1}\right)\\ d2\left(ur_{2}+ud_{2}\right)\\ d3\left(ur_{3}+ud_{3}\right) \end{array}\right). \end{array}$$

The auxiliary function *M*1, *M*2, *M*3 is defined as the following form:

(17) $$\begin{array}{c} M1\left(x_{1},x_{2},x_{3},y\;d,yd^{\left(1\right)},yd^{\left(2\right)},yd^{\left(3\right)},ud_{2},ud_{3}\right)\\ =N1\left(\cdot \right)+d1\left(u12\text{(}ur_{2}+ud_{2}\right)+u13\left(ur_{3}+ud_{3}\right),\\ M2\left(x_{1},x_{2},x_{3},y\;d,yd^{\left(1\right)},yd^{\left(2\right)},yd^{\left(3\right)},ud_{3}\right)\\ =N2\left(\cdot \right)+d2\left[u23\left(ur_{3}+ud_{3}\right)\right],\\ M3\left(x_{1},x_{2},x_{3},y\;d,yd^{\left(1\right)},yd^{\left(2\right)},yd^{\left(3\right)}\right)=N3\left(\cdot \right). \end{array}$$

By substituting (17) into (16), we can get

(18) $$\begin{array}{c} S^{-1}\left(x_{1},x_{2}\right){\dot{e}}_{3}\left(t\right)=\left(\begin{array}{c} M1\\ M2\\ M3 \end{array}\right)-\frac{1}{2}{\dot{S}}^{-1}\left(x_{1},x_{2}\right)e3+\left(\begin{array}{c} d1\left(ur_{1}+ud_{1}\right)\\ d2\left(ur_{2}+ud_{2}\right)\\ d3\left(ur_{3}+ud_{3}\right) \end{array}\right). \end{array}$$

By using neural network approximation, *M*1, *M*2, *M*3 can be written as

(19) $$\begin{array}{c} M1=\theta 1\phi 1^{T}\left(t\right)+\varepsilon 1,\\ M2=\theta 2\phi 2^{T}\left(t\right)+\varepsilon 2,\\ M3=\theta 3\phi 3^{T}\left(t\right)+\varepsilon 3. \end{array}$$

Among them, *θ*1^*T*^*θ*1 ≤ *θM*, *θ*2^*T*^*θ*2 ≤ *θM*, *θ*3^*T*^*θ*3 ≤ *θM*, |*ε*1| ≤ *ε*, |*ε*2| ≤ *ε*, |*ε*3| ≤ *ε*, and *θM* is unknown constant.

*ud*_*i*_ is designed as follows:

(20) $$\begin{array}{c} ud_{1}=-d1\hat{\theta }1\phi 1^{T}\left(t\right),\\ ud_{2}=-d2\hat{\theta }2\phi 2^{T}\left(t\right),\\ ud_{3}=-d3\hat{\theta }3\phi 3^{T}\left(t\right). \end{array}$$

By substituting (14), (19), and (20) into (18), the closed-loop error dynamics equation can be obtained:

(21) $$\begin{array}{c} S^{-1}\left(x_{1},x_{2}\right){\dot{e}}_{3}\left(t\right)\\ =\left(\begin{array}{c} \theta 1\phi 1^{T}\left(t\right)+\varepsilon 1\\ \theta 2\phi 2^{T}\left(t\right)+\varepsilon 2\\ \theta 3\phi 3^{T}\left(t\right)+\varepsilon 3 \end{array}\right)-\frac{1}{2}{\dot{S}}^{-1}\left(x_{1},x_{2}\right)e3-\left(\begin{array}{ccc} k_{1} & 0 & 0\\ 0 & k_{2} & 0\\ 0 & 0 & k_{3} \end{array}\right)e3-\left(\begin{array}{c} \hat{\theta }1\phi 1^{T}\left(t\right)\\ \hat{\theta }2\phi 2^{T}\left(t\right)\\ \hat{\theta }3\phi 3^{T}\left(t\right) \end{array}\right)\\ =\left(\begin{array}{c} \tilde{\theta }1\phi 1^{T}\left(t\right)\\ \tilde{\theta }2\phi 2^{T}\left(t\right)\\ \tilde{\theta }3\phi 3^{T}\left(t\right) \end{array}\right)+\left(\begin{array}{c} \varepsilon 1\\ \varepsilon 2\\ \varepsilon 3 \end{array}\right)-\frac{1}{2}{\dot{S}}^{-1}\left(x_{1},x_{2}\right)e3-\left(\begin{array}{ccc} k_{1} & 0 & 0\\ 0 & k_{2} & 0\\ 0 & 0 & k_{3} \end{array}\right)e3. \end{array}$$

The proposed Lyapunov candidate function is defined as follows:

(22) $$\begin{array}{c} V=\frac{1}{2}e1^{T}e1+\frac{1}{2}e2^{T}e2+\frac{1}{2}e3^{T}S^{-1}\left(x_{1},x_{2}\right)e3\\ +\frac{1}{2}\tilde{\theta }1^{T}\Gamma 1^{-1}\tilde{\theta }1+\frac{1}{2}\tilde{\theta }2^{T}\Gamma 2^{-1}\tilde{\theta }2+\frac{1}{2}\tilde{\theta }3^{T}\Gamma 3^{-1}\tilde{\theta }3. \end{array}$$

By deriving the Lyapunov function, the following equation can be obtained:

(23) $$\begin{array}{c} \dot{V}=-2e^{2}{}_{1,i}+e^{T}{}_{1,i}e_{2,i}-2e^{2}{}_{2,i}+e^{T}{}_{2,i}e_{3,i}+e3S^{-1}\left(x_{1},x_{2}\right){\dot{e}}_{3}\left(\text{t}\right)\\ -\tilde{\theta }1^{T}\Gamma 1^{-1}\dot{\hat{\theta }}1-\tilde{\theta }2^{T}\Gamma 2^{-1}\dot{\hat{\theta }}2-\tilde{\theta }3^{T}\Gamma 3^{-1}\dot{\hat{\theta }}3+\frac{1}{2}e3^{T}{\dot{S}}^{-1}\left(x_{1},x_{2}\right)e3. \end{array}$$

By substituting (21) into (23), according to the triangle inequality theorem, (23) can be transformed into the following form:

(24) $$\begin{array}{c} \dot{V}\leq -2{\left\|e1\right\|}^{2}-2{\left\|e2\right\|}^{2}+\frac{1}{2}{\left\|e1\right\|}^{2}+{\left\|e2\right\|}^{2}+1/2{\left\|e3\right\|}^{2}\\ +e3\left(\left(\begin{array}{c} \phi 1^{T}\left(t\right)\tilde{\theta }1+\varepsilon 1\\ \phi 2^{T}\left(t\right)\tilde{\theta }2+\varepsilon 2\\ \phi 3^{T}\left(t\right)\tilde{\theta }3+\varepsilon 3 \end{array}\right)-\frac{1}{2}{\dot{S}}^{-1}\left(x_{1}x_{2}\right)e3-\left(\begin{array}{ccc} k_{1} & 0 & 0\\ 0 & k_{2} & 0\\ 0 & 0 & k_{3} \end{array}\right)e3\right)\\ -\tilde{\theta }1^{T}\Gamma 1^{-1}\dot{\hat{\theta }}1-\tilde{\theta }2^{T}\Gamma 2^{-1}\dot{\hat{\theta }}2-\tilde{\theta }3^{T}\Gamma 3^{-1}\dot{\hat{\theta }}3+\frac{1}{2}e3^{T}{\dot{S}}^{-1}\left(x_{1},x_{2}\right)e3. \end{array}$$

Next, the adaptive update rate $\dot{\hat{\theta }}1,\dot{\hat{\theta }}2,\dot{\hat{\theta }}3$ of neural network weights is designed as

(25) $$\begin{array}{c} \dot{\hat{\theta }}1=\Gamma 1\phi 1\left(t\right)e3,1-\Gamma 1\eta 1\hat{\theta }1,\\ \dot{\hat{\theta }}2=\Gamma 2\phi 3\left(t\right)e3,2-\Gamma 2\eta 2\hat{\theta }2,\\ \dot{\hat{\theta }}3=\Gamma 3\phi 3\left(t\right)e3,3-\Gamma 3\eta 3\hat{\theta }3. \end{array}$$

By substituting the adaptive update rate into the original inequality (25), after expansion, we can get

(26) $$\begin{array}{c} \dot{V}\leq -\frac{3}{2}{\left\|e1\right\|}^{2}-{\left\|e2\right\|}^{2}+\frac{1}{2}{\left\|e3\right\|}^{2}\\ +e3\left(\left(\begin{array}{c} \phi 1^{T}\left(t\right)\tilde{\theta }1+\varepsilon 1\\ \phi 2^{T}\left(t\right)\tilde{\theta }2+\varepsilon 2\\ \phi 3^{T}\left(t\right)\tilde{\theta }3+\varepsilon 3 \end{array}\right)-\frac{1}{2}{\dot{S}}^{-1}\left(x_{1},x_{2}\right)e3-\left(\begin{array}{c} -\left(k1\right)e3,1\\ -\left(k2\right)e3,2\\ -\left(k3\right)e3,3 \end{array}\right)\right)\\ -\tilde{\theta }1^{T}\Gamma 1^{-1}\left(\Gamma 1\phi 1\left(t\right)e3,1-\Gamma 1\eta 1\hat{\theta }1\right)-\tilde{\theta }2^{T}\Gamma 2^{-1}\left(\Gamma 1\phi 1\left(t\right)e3,1-\Gamma 1\eta 1\hat{\theta }1\right)\\ -\tilde{\theta }3^{T}\Gamma 3^{-1}\left(\Gamma 1\phi 1\left(t\right)e3,1-\Gamma 1\eta 1\hat{\theta }1\right)+\frac{1}{2}e3^{T}{\dot{S}}^{-1}\left(x_{1},x_{2}\right)e3. \end{array}$$

After merging similar items, it is further transformed into the following form:

(27) $$\begin{array}{c} \dot{V}\leq -\frac{3}{2}{\left\|e1\right\|}^{2}-{\left\|e2\right\|}^{2}+\frac{1}{2}{\left\|e3\right\|}^{2}+e3,1\phi 1^{T}\left(t\right)\tilde{\theta }1+e3,2\phi 2^{T}\left(t\right)\tilde{\theta }2\\ +e3,3\phi 3^{T}\left(t\right)\tilde{\theta }3+e3,1\varepsilon 1+e3,2\varepsilon 2+e3,3\varepsilon 3-\left(k1\right)e3,1^{2}\\ -\left(k2\right)e3,2^{2}-\left(k3\right)e3,3^{2}-\tilde{\theta }1^{T}\phi 1\left(t\right)e3,1+\tilde{\theta }1^{T}\eta 1\hat{\theta }1\\ -\tilde{\theta }2^{T}\phi 2\left(t\right)e3,2+\tilde{\theta }2^{T}\eta 2\hat{\theta }2-\tilde{\theta }3^{T}\phi 3\left(t\right)e3,3+\tilde{\theta }3^{T}\eta 3\hat{\theta }3. \end{array}$$

Inequality (27) is reduced to the following form:

(28) $$\begin{array}{c} \dot{V}\leq -\frac{3}{2}{\left\|e1\right\|}^{2}-{\left\|e2\right\|}^{2}+\frac{1}{2}{\left\|e3\right\|}^{2}+e3,1\varepsilon 1+e3,2\varepsilon 2+e3,3\varepsilon 3-\left(k1\right)e3,1^{2}\\ -\left(k2\right)e3,2^{2}-\left(k3\right)e3,3^{2}+\tilde{\theta }1^{T}\eta 1\hat{\theta }1+\tilde{\theta }2^{T}\eta 2\hat{\theta }2+\tilde{\theta }3^{T}\eta 3\hat{\theta }3. \end{array}$$

At the same time, inequality scaling is performed on the residual term of the neural network approximation and the approximate value of the neural network weight, and the Lyapunov function inequality can be obtained as follows:

(29) $$\begin{array}{c} \dot{V}\leq -\frac{3}{2}{\left\|e1\right\|}^{2}-{\left\|e2\right\|}^{2}+\frac{1}{2}{\left\|e3\right\|}^{2}+\frac{1}{2}\varepsilon_{1}^{2}+\frac{1}{2}\varepsilon_{2}^{2}+\frac{1}{2}\varepsilon_{3}^{2}\\ +\tilde{\theta }1^{T}\eta 1\hat{\theta }1+\tilde{\theta }2^{T}\eta 2\hat{\theta }2+\tilde{\theta }3^{T}\eta 3\hat{\theta }3. \end{array}$$

Considering $\left\|\theta i\right\|\leq \theta M,\left\|\varepsilon i\right\|\leq \varepsilon M,\left\|\tilde{\theta }i\right\|\leq \tilde{\theta }M,i=1,2,3,$, we set *ki* − 1/2 > 1/2, and it can be derived from the original inequality:

(30) $$\begin{array}{c} \dot{V}\leq -\frac{3}{2}{\left\|e1\right\|}^{2}-{\left\|e2\right\|}^{2}-{\left\|e3\right\|}^{2}+\frac{1}{2}\varepsilon_{1}^{2}+\frac{1}{2}\varepsilon_{2}^{2}+\frac{1}{2}\varepsilon_{3}^{2}+\frac{1}{2}\eta_{1}^{2}\theta_{1}^{2}\\ +\frac{1}{2}\eta_{2}^{2}\theta_{2}^{2}+\frac{1}{2}\eta_{3}^{2}\theta_{3}^{2}-{\tilde{\theta }}_{1}^{T}\eta 1\tilde{\theta }1-{\tilde{\theta }}_{2}^{T}\eta 2\tilde{\theta }2-{\tilde{\theta }}_{3}^{T}\eta 3\tilde{\theta }3. \end{array}$$

We assume that the maximum eigenvalue of *S*^−1^(*x*_1_, *x*_2_) is less than a constant *λ*, and the constant *λ* is greater than zero. The matrix *S*(*x*_1_, *x*_2_) itself is a positive definite symmetric matrix with uniform positive definiteness. Therefore,

(31) $$\begin{array}{c} \dot{V}\leq -{\left\|e1\right\|}^{2}-{\left\|e2\right\|}^{2}-{\left\|e3\right\|}^{2}-{\tilde{\theta }}_{1}^{T}\eta 1\tilde{\theta }1-{\tilde{\theta }}_{2}^{T}\eta 2\tilde{\theta }2-{\tilde{\theta }}_{3}^{T}\eta 3\tilde{\theta }3+C. \end{array}$$

Among them, *C*=1/2*ε*_1_^2^+1/2*ε*_2_^2^+1/2*ε*_3_^2^+1/2*η*_1_^2^*θ*_1_^2^+1/2*η*_2_^2^*θ*_2_^2^+1/2*η*_3_^2^*θ*_3_^2^.

(32) $$\begin{array}{c} V\leq +\frac{1}{2}\left({\left\|e1\right\|}^{2}+{\left\|e2\right\|}^{2}+\lambda {\left\|e3\right\|}^{2}+{\tilde{\theta }}_{1}^{T}\Gamma 1^{-1}\tilde{\theta }1+{\tilde{\theta }}_{2}^{T}\Gamma 2^{-1}\tilde{\theta }2+{\tilde{\theta }}_{3}^{T}\Gamma 3^{-1}\tilde{\theta }3\right). \end{array}$$

According to the law of comparison, we have

(33) $$\begin{array}{c} \dot{V}\leq \frac{-2\min\left(1,\eta 1,\eta 2,\eta 3\right),}{\max\left(1,\lambda ,\Gamma 1^{-1},\Gamma 2^{-1},\Gamma 3^{-1}\right)}V+C. \end{array}$$

By solving the differential inequality, we can get

(34) $$\begin{array}{c} V\leq -\frac{c}{m}+\left(V\left(0\right)+\frac{c}{m}\right)e^{mt}. \end{array}$$

Among them, *m*=−2min(1, *η*1, *η*2, *η*3, )/max(1, *λ*, Γ1^−1^, Γ2^−1^, Γ3^−1^).

Therefore,

(35) $$\begin{array}{c} -\frac{c}{m}{\left\|e\right\|}^{2}\leq V\leq -\frac{c}{m}+\left(V\left(0\right)+\frac{c}{m}\right)e^{mt}. \end{array}$$

Thus, it is indicated that the tracking error *e*1, *e*2, *e*3 and the weight estimation error index $\tilde{\theta }1,\tilde{\theta }2,\tilde{\theta }3$ converge to zero.

## 4. Tourism Demand Forecast Based on Adaptive Neural Network Technology

**Figure 1:**
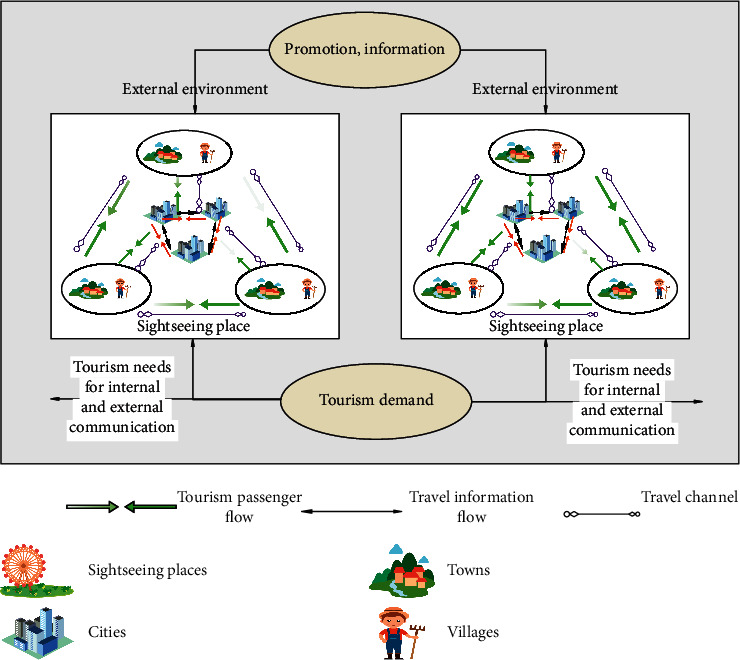
Spatial system diagram of tourist destinations.

In order to respond to the spatial increase of local tourism demand status, the short-sighted path dependence of the unidirectional flow view and the static destination market concept should be eliminated. Moreover, it is necessary to reconstruct a new tourist destination spatial system with both destination and tourist source functions from a more microscopic perspective (see Figure 1). The spatial reorganization of tourist destinations is explored under the continuous deepening of small- and medium-scale tourism spatial behavior. The system should be a specific organic whole composed of several interacting and interdependent tourism elements within a certain geographical space. It uses the existing borders of jurisdictions as the dividing lines between local and remote areas. This includes not only the part of the spatial system formed by the inflow of tourist from outside the region involved in the original space system into the tourist destination, but also the part of the regional tourism space system such as cities, towns, and rural areas. At the same time, it takes tourist destinations that have both the functions of tourist source and destination as the main body to form a space system with specific order and complementary internal and external space with factors such as regional tourism environment, tourism channels, tourism information, and tourism flow. It needs to be pointed out that the reconstruction of the new tourist destination space system is not a complete denial of the original tourist destination space system, but a supplement and improvement to its system.

**Figure 2:**
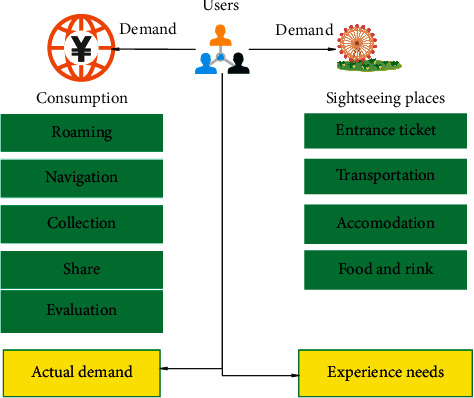
Analysis of user needs.

When designing the system, the main direction is around the surrounding information of the scenic spot. For example, we need to add functions that allow users to search for, roam, navigate, collect, share, and pay for scenic spots to facilitate users to screen scenic spots and provide the readability of tourism quality and the convenience of transportation. In the process of searching and roaming at scenic spots, tourists can find relevant information such as the ticket limit of scenic spots, travel, and transportation costs. In addition, it should be noted that the platform must take into account both the emotional needs and consumer needs of users. On the one hand, after users have used the travel tools provided by the platform and browsed the target attractions, they will need to communicate with other people on the Internet. On the other hand, users also need to store and classify related information after using the platform. Therefore, we also need to provide a storage and classification function, so that users can save time when using the platform for the second time and quickly find the previously stored scenic spot information. The analysis of user needs is shown in Figure 2.

**Figure 3:**
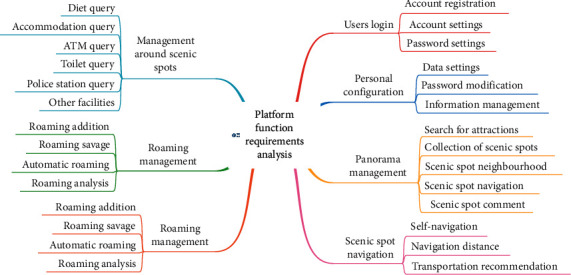
Analysis of platform function requirements.

The analysis of platform function requirements is shown in Figure 3.

**Figure 4:**
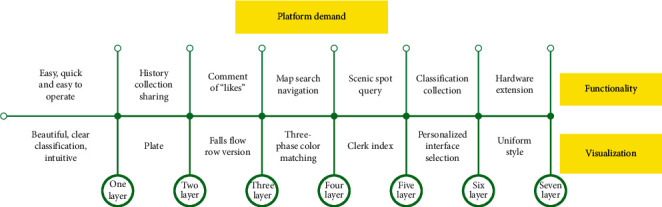
Fishbone diagram of the analysis of platform functional requirements.

Before planning a trip, users need to search for resources effectively. It is necessary to classify the platform's attractions resources, and through the history function, users can easily call up the browsing history. Secondly, after the user has screened the destination, the user needs to understand the surroundings. At the same time, it is necessary to prompt peripheral information such as toilets, ATMs, and hotels during the virtual roaming process. When the platform constructs the framework, it needs to introduce data about city-related information. After the user has searched the surrounding information of the destination, the user needs to pay for the services that can be provided on the Internet. Therefore, the platform needs to add the function of Internet payment. After the travel experience, there must be follow-up emotional expressions, so the platform needs to have a comment function. The fishbone diagram of the platform function requirement analysis is shown in Figure 4.

**Figure 5:**
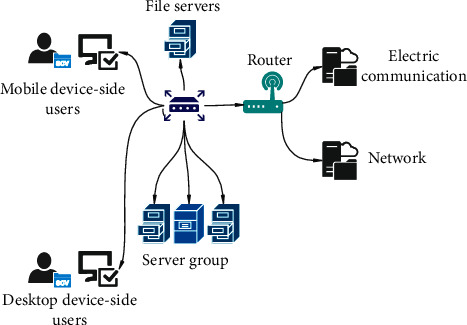
Network topology diagram.

The terminals involved in this paper include mobile devices and desktop devices. Mobile ports include tablet computers and mobile phones, and desktop devices are computer hosts. The network topology is shown in Figure 5.

Space, attribute, and time are the three basic characteristics of geographic phenomena and the three basic elements of geographic analysis. Time information is helpful to explore the movement laws of individual activities and predict future development trends. However, due to the lack of effective analysis methods for massive spatiotemporal data and other reasons, previous studies usually ignore time information. However, time geography introduces “time” and uses the concept of “time and space” to simultaneously analyze the three types of information of time, space, and attributes within the same framework, which bridges the traditional geography's neglect of time. It should be noted that time geography is not the geography of time, but a methodology for studying the relationship between the human behavior and the objective space-time environment. Temporal geography is the geography that studies the temporal and spatial characteristics of individual behavior under various constraints. It believes that the space-time range of individual activities is restricted by a variety of factors in itself and its surrounding environment, which can be summarized into three aspects: ability restriction, combination restriction, and authority restriction. At the same time, time geography borrows the concept of “space-time prism” to describe the space-time area in which individuals can carry out activities under various constraints. The space-time prism image represents the various possibilities of individual activities. The upper and lower vertices of the prism are determined by the time point, and the boundary of the prism is determined by the conditions of transportation.

**Figure 6:**
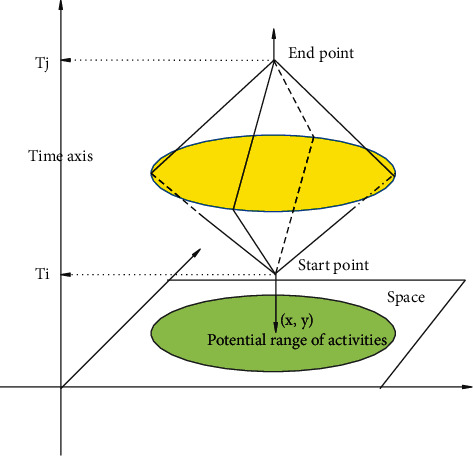
Schematic diagram of a space-time prism with the start and end points at the same spatial position (three-dimensional).

**Figure 7:**
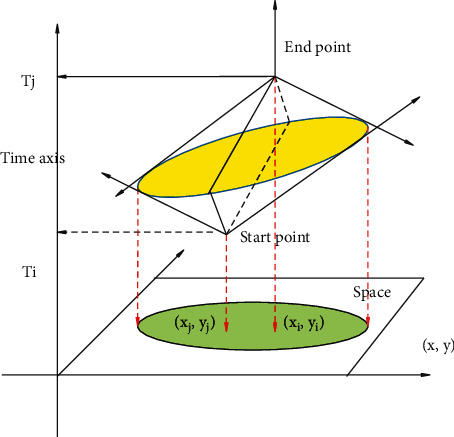
Schematic diagram of space-time prisms with start and end points at different spatial positions (three-dimensional).

With the quantitative development of time geography, probabilistic time geography began to appear and attracted attention. Although classical time geography borrows space-time prisms to express the uncertainty of individual movement in space-time regions, it assumes that individual objects are evenly distributed within the reach. However, according to the first theorem of geography, it can be known that the possibility of individual moving objects in different reachable positions is not always uniform. Probabilistic time geography is based on this idea. It is believed that the possibility of moving objects distributed in each reachable position is not always equal, but is distributed within the reachable range with a certain probability value. Moreover, this probability value continues to change over time. Probabilistic time geography advocates the use of probabilistic methods to describe the nonequal possibilities of the reachable locations of mobile objects, which is an extension of classical time geography based on probability. Based on the probability characteristics of variance and other probabilities shown by time geography of different probability models, a time geography probability model of directional movement is constructed based on the Brown Bridge probability and the total probability formula, respectively. Probabilistic time geography is still immature and needs further improvement. However, the idea that the analysis of individual moving objects needs to consider the actual probability distribution provides a theoretical source for this paper to use the probability method to predict the temporal and spatial distribution of tourists in scenic spots. The schematic diagram of the space-time prism with the starting point and the end point in the same spatial position is shown in Figure 6, and the schematic diagram of the space-time prism with the starting point and the end point in different spatial positions is shown in Figure 7.

**Figure 8:**
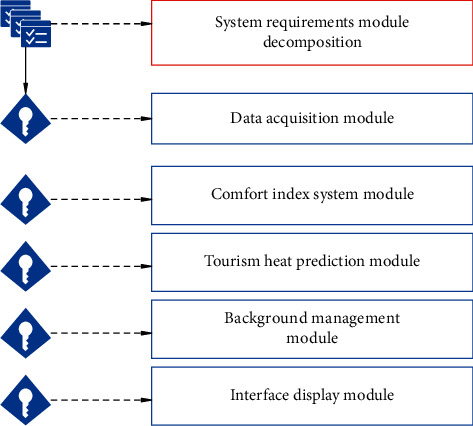
Functional decomposition of system requirements.

This paper mainly studies tourism demand forecast based on adaptive neural network and realizes the heat forecast of tourist attractions by fusing the comfort index and specific adaptive neural network forecast algorithms. Moreover, this paper applies the research results to specific engineering practice and establishes a tourist attraction heat forecast system based on the comfort index. The establishment of the system can help tourists better understand the comfort and heat information of a scenic spot, help tourists choose scenic spots reasonably, and also benefit enterprises' decision-making on tourism projects, as well as their recommendation and management of tourists. The system should have three major functions: data collection, data calculation and analysis, and data management and display. The detailed functional modules are data collection module, comfort index system module, tourism popularity forecast module, background management module, and result display module. The five major functional modules of the system are shown in Figure 8.

The data acquisition module mainly realizes data acquisition and data preprocessing. The data collection module performs related collection work according to different data requirements. After collection, the data is saved and backed up to the relevant server database, and a data interface is provided to enable other modules to use the travel data. The data collection module should be able to automatically obtain tourist data, weather data, environmental pollution data, road conditions around the scenic spot, etc. according to the configuration. The data acquisition module must have certain data preprocessing capabilities and realize data cleaning and data integration work through programming. The data acquisition module must have a certain degree of anti-interference ability and response speed and provide multithreaded data acquisition and processing capabilities. The configuration administrator can view all the scenic spot information that the system can collect and the scenic spot information currently collecting data and can add and delete the scenic spots collected by the data collection module. When adding tourist attractions to the collection module, the configuration manager can set the time range and time interval for tourism data collection. When the configuration administrator deletes or adds tourist attractions to the data collection module, the delay will take effect until the next day. After adding the scenic spots where the data collection module works, the system will query all the URLs of the current scenic spots that collect data by default. The configuration administrator needs to confirm whether all the scenic spots URLs are valid. If they are invalid, they need to be added manually. At the same time, the data collection module requires a built-in web page analysis program. The previous analysis needs are only for China Weather Network, Green Breathing Network, Beijing Meteorological Bureau Network, Beijing Tourism Network, and Nanjing Tourism Network. When confirming the URL of the scenic spot, we need to ensure that the system can parse the configured URL. At the same time, the configuration administrator needs to configure the collection of API interface information around the scenic spot to ensure accurate acquisition of the road situation around the scenic spot.

**Figure 9:**
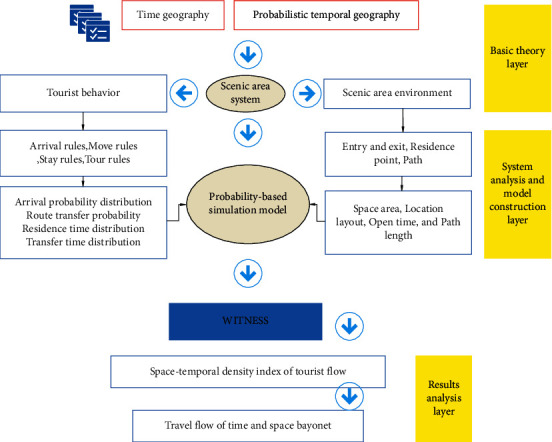
Forecast model of travel time and space bayonet.

This paper integrates time geography and probability time geography theory, system analysis theory, and travel flow spatiotemporal bayonet theory and proposes a research framework based on WITNESS simulation system to predict travel flow spatiotemporal bayonet. Among them, time geography and probabilistic time geography provide a theoretical basis for establishing a simulation model of a scenic spot system based on probability. According to the system analysis theory, the scenic spot system is analyzed from the two aspects of scenic spot environment and tourist behavior rules, and the scenic spot environment is abstracted and simplified into three categories: entrances and exits, paths, and stop points. The tourist behavior rules are divided into tourist arrival rules, mobile stay rules, and tour rules. According to the results of the system analysis, data on the area of the scenic area, location layout, path length, tourist arrival probability distribution, route transition probability, stay time probability distribution, and travel time of scenic spots are collected. These data provide initial parameters for the probability-based simulation system model, and the model is implemented on the WITNESS simulation software platform. Finally, according to the theory of travel flow time and space bayonet, it is proposed to use the time and space density index of tourism flow as an index to identify the time and space bayonet of tourism flow. The proposed research framework can provide reference and guidance for establishing the same simulation model for other types of scenic spots to predict the spatiotemporal bayonet of tourism flows, as shown in Figure 9.

**Figure 10:**
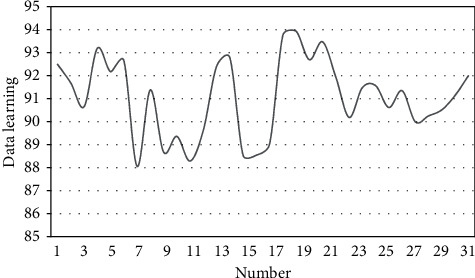
Statistical diagram of the processing effect of spatiotemporal tourism data.

After constructing a tourism demand forecast model based on adaptive neural network technology, the performance of this model is verified. Based on actual data, this paper uses the simulation platform proposed in this paper to process the time-space travel data, analyze the data processing effect, and obtain the results shown in Table 1 and Figure 10.

**Figure 11:**
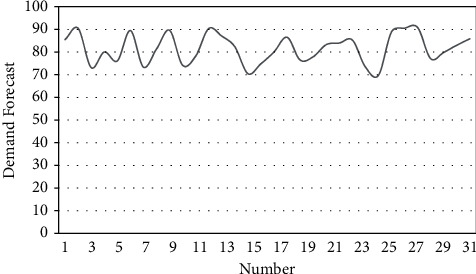
Statistical diagram of tourism demand forecast.

From the above analysis, it can be seen that the tourism demand forecast model based on adaptive neural network technology proposed in this paper has good spatiotemporal tourism data processing effects. After that, the tourism demand forecast model based on adaptive neural network technology is evaluated for the effect of tourism demand forecast, and the results shown in Table 2 and Figure 11 are obtained.

From the above research, it can be seen that the tourism demand forecast model based on adaptive neural network technology proposed in this paper performs well in tourism demand forecast and meets the actual demand of modern tourism forecast.

## 5. Conclusion

The study of factors affecting tourism demand is the key content of tourism demand forecasting. At present, domestic and foreign researches on this aspect have formed a more systematic theory. Quantitative analysis method is currently widely used in foreign countries to study the factors affecting tourism demand. Economic factors can be divided into income, price, leisure time, and marketing. Income includes the personal disposable income and the GDP of the source area; price includes the transportation expenses, the price of products and reading materials in tourist destinations, the price level of competing destinations, and the exchange rate. Noneconomic factors include consumers' personal preferences, consumption habits, local political factors, travel restrictions, and other social and cultural factors. This article uses adaptive neural network technology to forecast and analyze tourism demand, build a business intelligence model, and improve the prediction effect of subsequent tourism demand. The experimental research results show that the tourism demand prediction model based on adaptive neural network technology proposed in this paper performs well in tourism demand prediction and meets the actual demand of modern tourism prediction.

## Figures and Tables

**Table 1:** Processing effect of spatiotemporal tourism data.

Number	Data learning
1	92.51
2	91.70
3	90.67
4	93.20
5	92.19
6	92.67
7	88.08
8	91.39
9	88.70
10	89.38
11	88.31
12	89.65
13	92.42
14	92.79
15	88.56
16	88.56
17	89.05
18	93.77
19	93.95
20	92.71
21	93.49
22	91.95
23	90.20
24	91.49
25	91.58
26	90.63
27	91.36
28	90.01
29	90.27
30	90.53
31	91.16
32	92.01

**Table 2:** Results of tourism demand forecast.

Number	Demand forecast
1	85.49
2	90.43
3	73.12
4	80.01
5	76.08
6	89.47
7	73.34
8	81.27
9	89.60
10	74.15
11	78.18
12	90.35
13	87.01
14	82.31
15	70.46
16	74.78
17	80.01
18	86.49
19	76.47
20	77.90
21	83.25
22	83.95
23	85.27
24	73.40
25	69.73
26	88.87
27	90.52
28	90.80
29	77.06
30	79.81
31	83.01
32	85.81

## Data Availability

The labeled datasets used to support the findings of this study are available from the corresponding author upon request.
